# Acute effect of a cod protein hydrolysate on postprandial acylated ghrelin concentration and sensations associated with appetite in healthy subjects: a double-blind crossover trial

**DOI:** 10.29219/fnr.v63.3507

**Published:** 2019-10-22

**Authors:** Hanna Fjeldheim Dale, Caroline Jensen, Trygve Hausken, Einar Lied, Jan Gunnar Hatlebakk, Ingeborg Brønstad, Dag Arne Lihaug Hoff, Gülen Arslan Lied

**Affiliations:** 1 Centre for Nutrition, Department of Clinical Medicine, University of Bergen, Bergen, Norway; 2 Division of Gastroenterology, Department of Medicine, Haukeland University Hospital, Bergen, Norway; 3 National Centre of Functional Gastrointestinal Disorders, Haukeland University Hospital, Bergen, Norway; 4 Firmenich Bjørge Biomarin AS, Ellingsøy, Ålesund, Norway; 5 Department of Clinical Medicine, University of Bergen, Bergen, Norway; 6 National Centre for Ultrasound in Gastroenterology, Haukeland University Hospital, Bergen, Norway; 7 Division of Gastroenterology, Department of Medicine, Ålesund Hospital, Møre & Romsdal Hospital Trust, Ålesund, Norway; 8 Department of Clinical and Molecular Medicine, Faculty of Medicine and Health Sciences, Norwegian University of Science and Technology, Trondheim, Norway

**Keywords:** hunger, overweight, marine peptides, gastric hormones, nutrition supplement

## Abstract

**Background:**

Fish protein hydrolysates are suggested to contain bioactive sequences capable of affecting metabolic pathways involved in the regulation of glucose metabolism and body weight when consumed in low doses. Modulation of the appetite-regulating hormone ghrelin may explain suppression of insulin secretion and weight loss observed in previous studies with fish protein hydrolysates.

**Objective:**

This study aimed to assess the effect of a single, low dose of cod protein hydrolysate (CPH) before a breakfast meal on postprandial acylated ghrelin concentration and sensations associated with appetite in healthy subjects.

**Design:**

In this explorative trial with a crossover design, 41 healthy individuals (15 males and 26 females, age 51 ± 6 years) completed 2 study days separated by 4–7 days of washout. On both study days, a test drink containing 20 mg CPH or casein (control) per kg body weight was given immediately before a standardized breakfast meal. Acylated ghrelin concentrations were measured before test drink/breakfast (baseline) and at time 0, 20, 40, 80, and 180 min postprandially. Sensations associated with appetite were measured by a Visual Analog Scale (100 mm) at baseline and 0, 20, 40, and 180 min postprandially.

**Results:**

Statistically, no difference was observed between CPH and control for postprandial acylated ghrelin concentrations (mean difference geometric mean: 1.05 pg/mL, 95% confidence interval [CI]: 0.97–1.13, *P* = 0.266), or between the total area under the curve (tAUC) for acylated ghrelin after CPH (tAUC = 17518 pg/mL × min, 95% CI: 0–47941) and control (tAUC = 17272 pg/mL × min, 95% CI: 0–48048, *P* = 0.991). No differences were found between CPH and control for sensation of appetite, according to tAUC of postprandial scores for satiety (*P* = 0.794) and the feeling of fullness (*P* = 0.996).

**Conclusion:**

We did not find an effect of a single dose of CPH on postprandial concentrations of acylated ghrelin or sensations related to feeling of hunger, compared to control. Further studies should aim to evaluate the effect of a supplement with CPH given daily over a period of time.

## Popular scientific summary

- Ghrelin is an appetite-regulating hormone, with high concentrations before a meal and reduced concentrations after a meal. Compounds with the ability to suppress the action of ghrelin may be valuable for weight regulation.
- Fish protein hydrolysates are suggested to contain bioactive peptides capable of affecting glucose metabolism and body weight.
- In this study, no effect of a supplement with cod protein hydrolysate on postprandial ghrelin concentrations or sensations related to appetite was observed.

Ghrelin is a gastric hormone, capable of stimulating hunger and influence energy homeostasis (1). It is a small peptide consisting of 28 amino acids, secreted from neuroendocrine cells in the submucosal layer of the stomach (2). The circulating ghrelin concentration gradually increases before a meal and decreases with feeding (3). Two forms of ghrelin are present in the circulation, acylated and non-acylated ghrelin, of which the acylated form is the one known to activate the ghrelin receptor (2).

Acylated ghrelin is a natural ligand binding to the growth hormone secretagogue (GHS) receptor, leading to stimulation of the secretion of growth hormone (GH), reduction in insulin secretion and glucose tolerance (4–6). Furthermore, it has been shown that acylated ghrelin holds potent adipogenic and orexigenic effects mediated through the GHS receptor located in the central nervous system (CNS) (4). Acylated ghrelin is known to directly activate pathways in the CNS controlling both parasympathetic and sympathetic nerve activity through GHS receptors (7) and possibly indirectly suppresses insulin secretion via neural signaling (8). These qualities have created the idea that compounds having the ability to suppress the action of ghrelin may be valuable for the prevention or treatment of overweight, obesity, insulin resistance, and abnormal lipid and glucose metabolism (9, 10).

In previous studies in rats and humans, it has been observed that the intake of low doses of peptides from fish is capable of beneficially influencing glucose metabolism (11–14), reducing adipose tissue mass, and improving serum fatty acid composition (15, 16), when compared to placebo or casein. In addition, some recent studies have found fish protein hydrolysates to beneficially influence hormones involved in the regulation of appetite, such as cholecystokinin (CCK) and glucagon-like peptide 1 (GLP-1), as well as influence the subjective feeling of craving sweets (17, 18), when compared to placebo in human subjects. The suggested effective daily dose based on the current literature in human subjects ranges from 1 to 6 g per day (13, 18). Consequently, the hypothesized effect of a fish protein hydrolysate is not due to the consumed protein *per se,* which is negligible compared to the normal recommended total daily dietary intake of a healthy individual (e.g. 65–80 g protein per day with body weight 80 kg) (19). A possible mechanism for suppression of postprandial insulin concentration and weight loss could be modulation of postprandial ghrelin concentrations, and a low dose of fish protein hydrolysate is presumed to be effective due to the content of bioactive peptides with unique amino acid sequences (20). In the current trial, we hypothesize that a potential suppressing effect on appetite and postprandial ghrelin levels can be attributed to a high fraction of di- and tripeptides with branched-chain amino acids in the cod protein hydrolysate (CPH), facilitating rapidly absorption from the gastrointestinal tract and possibly capable of influencing pathways involved in the regulation of appetite.

As metabolism and energy expenditure decrease with age, middle-aged individuals often experience weight gain. Thus, middle-aged individuals might benefit from an intervention targeting appetite and hunger regulation. Data on the specific effect of a dietary supplement with a fish protein hydrolysate on ghrelin concentrations and sensations associated with appetite have, to our knowledge, previously not been published. The present study aimed to assess the effect of a single, low dose of CPH before a breakfast meal on postprandial acylated ghrelin concentration and sensations associated with appetite in healthy, middle-aged to elderly subjects.

## Material and methods

Data on subjects and methods have been described in detail in a previous publication (14).

### Trial design

The study was a double-blind crossover trial, which included two study visits for each subject, with 4–7 days of washout in between. The intervention included serving of a test drink containing 20 mg of CPH per kg body weight (test material) or control (casein) in randomized order, immediately before a standardized breakfast meal was served. The CPH and casein powder were mixed with cold water and served as a drink. The primary outcome of the intervention (postprandial response in glucose metabolism) is reported in a previous publication (14). Here, we report the secondary outcome: acylated ghrelin concentrations measured for 180 min postprandially and subjective sensations associated with appetite.

This study was conducted according to the guidelines laid down in the Declaration of Helsinki, and all procedures were approved by the Regional Committees for Medical and Health Research Ethics of Central Norway (2017/1794). Written informed consent was obtained from all subjects. The trial was registered at clinicaltrials.gov as NCT03669796.

### Participants

Subjects were recruited at Haukeland University Hospital and Ålesund Hospital between October 2017 and February 2018. Potential subjects were interviewed for general eligibility and compliance with inclusion and exclusion criteria by telephone. Candidates were invited for a further screening visit.

Inclusion criteria were as follows: age 40–65 years and body mass index (BMI) 20–30 kg/m^2^. Exclusion criteria were fish allergy, pharmacologically treated diabetes mellitus, elevated blood pressure, chronic diseases that might affect the evaluation of the study endpoints, and acute infections.

### Study protocol

The screening visit included a clinical examination by a physician, biochemistry tests for safety purposes (leukocyter, trombocytes, hemoglobin, fasting glucose, long-term blood glucose, C-reactive protein, creatinine, sodium, potassium, kidney function estimate, liver enzymes and muscle enzymes) and compliance with inclusion criteria, measuring of height, weight, and blood pressure, as well as assessment of the level of physical activity. The level of physical activity was assessed by asking the participants two questions regarding moderate physical activity and vigorous activity (self-reported). The participants were instructed not to change the diet composition or the level of physical activity during the study period. On the day preceding each study day, the participants received a standardized porridge evening meal to be eaten before 8:30 pm. After this, the subjects were instructed to fast until the next morning and were only allowed to drink water. On study days, the participants came to the research units in a fasting state between 08:00 am and 09:00 am. After the first blood sample, the subjects were served the test drink, before the breakfast meal was provided. Fifteen minutes after the breakfast was served, the first post-meal sample (0 min sample) was taken.

The standardized breakfast meal consisted of two slices of bread (50% whole wheat, 80 g bread), 10 g margarine, 20 g strawberry jam, and 20 g white cheese. This provided a total of 355 kcal (1,485 kJ, 41 g carbohydrate, 12.5 g protein, 15 g fat). The drink contained on average 35.9 g carbohydrate and 145 kcal (607 kJ). Thus, including the drink, the breakfast provided in total 500 kcal (2,092 kJ) and 77 g carbohydrate, equal at both study days. Water was given *ad libitum*, but no coffee or tea was allowed. The subjects spent 4 h in the research units, and repeated sampling of blood was conducted before serving of the test drink and breakfast, and at time 0, 20, 40, 80, and 180 min postprandially.

### Assessments

Assessment of medical history, measurement of biochemical variables and safety parameters were conducted before randomization. During the two study visits, acylated ghrelin was measured in plasma samples taken before serving of the test drink and breakfast (baseline), at time 0, 20, 40, 80, and 180 min postprandially. The subjects had 15 min to finish the breakfast before the 0 min sample was taken. Blood pressure was measured before intervention, after 40 and 180 min after the intervention, as a safety parameter.

Appetite sensation was assessed on a Visual Analog Scale (VAS) of 100 mm in length, addressing the feeling of fullness and satiety. The VAS questionnaire also included three questions regarding adverse gastrointestinal symptoms (pain, discomfort, and nausea). The VAS was filled out five times during the study visit, at baseline, time 0 and 20, 40, and 180 min after the breakfast meal. Additionally, a questionnaire validated for the evaluation of different gastrointestinal symptoms was filled out before the breakfast meal and at the end of each study day (21). The questionnaire assessed nausea, bloating, stomach pain, constipation, and diarrhea, as well as hunger/satiety with a score from 0 to 10, of which the score 10 indicated severe symptoms and being fully satiated.

### Test materials

The test material was a lemon-flavored powder provided from the manufacturer (Firmenich Bjørge Biomarin AS, Ålesund, Norway) in standardized bottles to be added 150 mL cold water. The powder contained 4% protein (CPH raw material or whole casein) and 96% carbohydrate (maltodextrin). Thorough laboratory tests assured that it was not possible to identify the active ingredient from the control, according to flavor or appearance. Each subject was given individually adjusted doses of 20 mg/kg body weight of CPH or casein. The drinks were made isonitrogenous to avoid bias due to difference in nitrogen content, and equal amounts of nitrogen in the form of casein were added to the control drink. Both drinks contained on average 1.6 g protein; thus it constituted only a small fraction of the total protein content of the standardized breakfast meal. Casein was chosen as the control as it has previously shown to not affect blood glucose or insulin sensitivity when compared with proteins from cod and soya (22). The casein used as control was present as whole protein and did not contain free amino acids or peptides. The production and composition of the test materials has been described in detail in previous publication (14).

### Analysis of blood samples

Samples of venous blood were repeatedly collected using an intravenous catheter from the antecubital vein. Samples were analyzed according to standard accredited methods at the Laboratory for Clinical Biochemistry, Haukeland University Hospital (Bergen, Norway) and the Department of Medical Biochemistry, Ålesund Hospital (Ålesund, Norway).

Plasma ghrelin was obtained by centrifugation of full blood using 4 mL anticoagulant (EDTA-K3)/Aprotinin blood collecting tubes (VACUETTE^®^, Greiner Bio One International GmbH, cat # 454261, Kremsmünster, Austria) at 1800 × *g* at 4°C for 10 min within 20 min after blood sampling. Plasma was stored frozen at −80°C prior to analysis. The ghrelin analyses were performed using Acylated Ghrelin-Easy Sampling Enzyme Immunoassay kit (Bertin Pharma, Montigny-le-Bretonneux, France, ref: #A11306).

### Statistical analysis

SPSS data package (SPSS Statistics 24.0, IBM Company, Armonk, NY, USA) and GraphPad Prism version 7.0 (GraphPad Software, Inc., San Diego, CA, USA) were used for statistical analysis. Shapiro–Wilk’s test was conducted to assess normal distribution of data. A multivariable, repeated-measures linear mixed-effects regression analysis (adjusted for BMI and gender) was conducted in SPSS in order to evaluate the difference between the concentrations of acylated ghrelin after CPH and control. The data for acylated ghrelin were non-normally distributed; thus, it was log-transformed before analysis and presented as log mean and back-transformed values (geometric means). Graphical work and total area under the curve (tAUC) analysis for acylated ghrelin concentrations and symptom VAS-scores were conducted in GraphPad. The difference in baseline and end-point scores for the additional questionnaire was evaluated by a paired *t*-test. Assessment of correlations was done with Pearson’s correlation coefficient. *P*-values < 0.05 were considered statistically significant.

The number of participants was not calculated according to a power analysis, due to lack of similar studies. Previous research reporting on effect of cod proteins in humans is based on whole fish (23) or long-term use of fish protein supplement (12, 13); thus, we did not find any data suitable as basis for power analysis for our design.

## Results

### Subjects

Seventy-eight subjects were screened for inclusion and exclusion criteria over telephone, of which 47 were enrolled to a screening visit. Six participants withdrew before the first study visit, and 41 participants completed the trial, of which 15 males and 26 females. The inclusion process has previously been described and illustrated (14). The mean age of the participants was 51 ± 6 years (range 40–64 years). The mean body weight of the participants was 77.3 ± 13.5 kg. Dependent on body weight, the subjects consumed CPH in a dose ranging from 1.2 to 2.3 g (mean 1.5 g). Mean BMI was 25.2 ± 3 kg/m^2^. Twenty-three participants had BMI ≤ 25 kg/m^2^, whereas 18 participants had BMI > 25 kg/m^2^. Baseline characteristics and comparison of gender distribution are presented in Table 1.

**Table 1:** Baseline characteristics of the 41 participants (26 females and 15 males) included in the study at the Haukeland University Hospital and Ålesund Hospital

Characteristics	Total subjects (*n* = 41)		Female (*n* = 26)		Male (*n* = 15)		*P*
	Mean	Standard deviation	Mean	Standard deviation	Mean	Standard deviation	
Age, years	51.0	6.0	52.1	6.2	49.0	5.0	0.104
Body weight	77.3	13.5	71.6	10.8	87.2	12.3	0.001
Body mass index, kg/m^2^	25.2	3.0	24.7	3.0	26.0	2.9	0.183
Acylated ghrelin, pg/mL	93.7	194.9	81.4	184.7	115.0	210.6	0.453

Baseline acylated ghrelin concentrations are merged values for the baseline value at both study visits.

### Acylated ghrelin concentrations

Mean fasting acylated ghrelin levels were higher before the CPH intervention than before the control intervention (97.4 ± 196.3 pg/mL vs. 90.0 ± 194.0 pg/mL, respectively), but the difference was not significant (*P* > 0.999). Suppression of acylated ghrelin (C_min_) was greatest: 80 min postprandially after CPH and 20 min postprandially after control, with a C_min_ mean difference from baseline of −14.0 ± 21.4 after CPH and −5.3 ± 22.8 after control, at these times, respectively (*P* = 0.681).

**Fig. 1:**
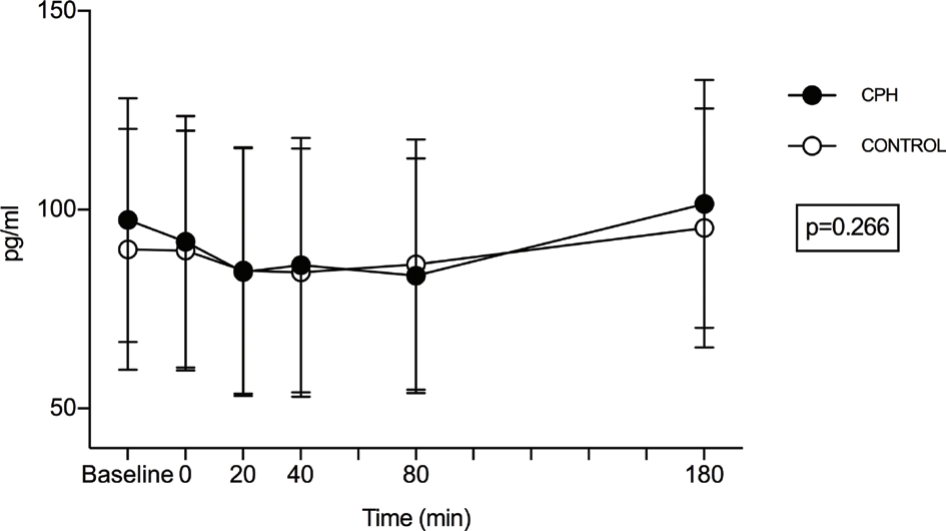
Metabolic response in acylated ghrelin concentration after intake of a standardized breakfast meal supplemented with a drink containing a cod protein hydrolysate (CPH) or control (casein). Results are presented for 41 healthy subjects. Time point 0 min shows values measured right after the intake of breakfast and test drink. Values are presented as mean + SD. Statistically, no differences were observed between CPH and control for acylated ghrelin concentration in a mixed-model regression analysis (*P* = 0.266).

Statistically, no differences were observed between CPH and control for postprandial acylated ghrelin concentration in a mixed-effects regression analysis (mean difference of the geometric mean: 1.05 pg/mL, 95% confidence interval [CI]: 0.97–1.13, *P* = 0.266) (Fig. 1). Additionally, no difference was observed between the tAUC for acylated ghrelin concentrations after CPH (tAUC = 17518 pg/mL × min, 95% CI: 0–47941) and control (tAUC = 17272 pg/mL × min, 95% CI: 0–48048, *P* = 0.991).

No correlation was observed between body weight (kg) and baseline concentration of acylated ghrelin (mean baseline value before CPH and control) (*r* = 0.118, *P* = 0.463). When adjusting for BMI and gender in the mixed-effects regression analysis, no differences were observed between subjects with BMI ≤ 25 kg/m^2^ compared to those with BMI > 25 kg/m^2^ (*P* = 0.681), or between genders (*P* = 0.627).

### Sensation of appetite

**Fig. 2:**
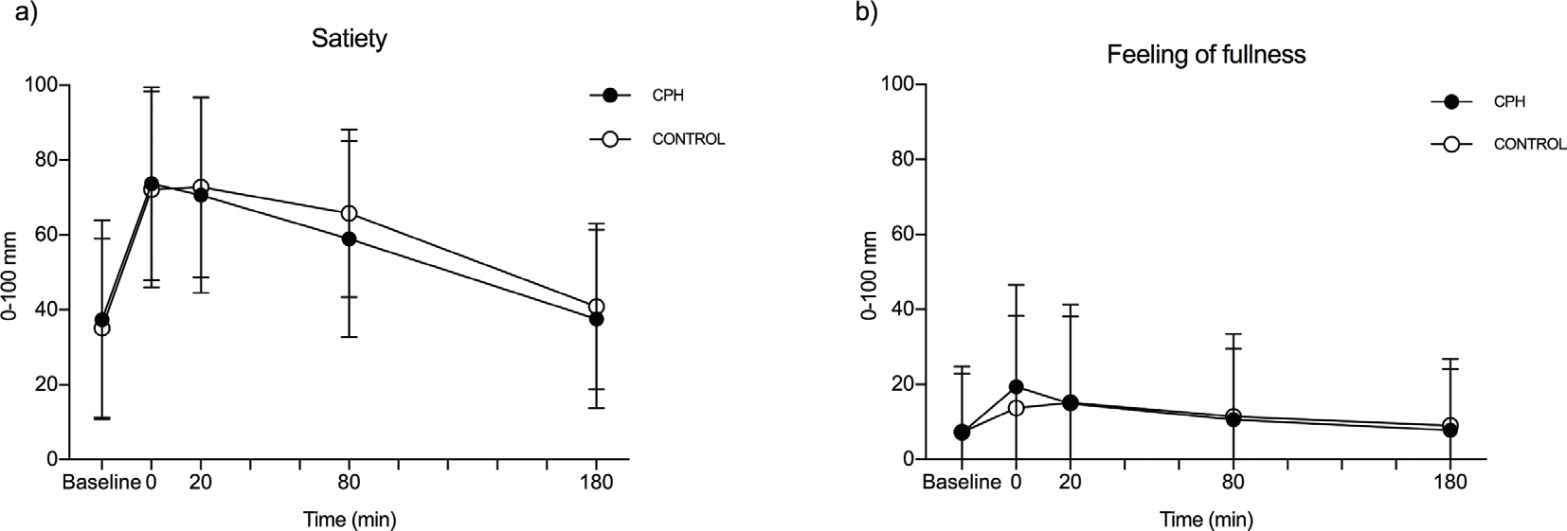
Symptom scores from a VAS-questionnaire addressing satiety (a) and the feeling of fullness (b) after intake of a standardized breakfast meal supplemented with a drink containing either a cod protein hydrolysate (CPH) or control (casein). Results are presented for 41 healthy subjects. Time point 0 min shows values measured right after the intake of breakfast and test material. Values are presented as mean + SD. Statistically, no differences were found between CPH and control for sensation of appetite, according to the tAUC of postprandial scores for satiety (*P* = 0.794) and the feeling of fullness (*P* = 0.966).

Baseline scores for satiety were numerically the same before each intervention (CPH: 37.4 ± 26.6, control: 35.1 ± 23.9, *P* = 0.997). No difference was observed between the tAUC for the postprandial satiety scores after CPH (tAUC = 10989 mm × min, 95% CI: 6794–15185) and control (tAUC = 11742 mm × min, 95% CI: 8001–15483, *P* = 0.794). Data are presented in Fig. 2a.

Baseline scores for the feeling of fullness were numerically the same before each intervention (CPH: 7.1 ± 15.7, control: 7.3 ± 17.5, *P* > 0.999). No difference was observed between the tAUC for the postprandial feeling of fullness scores after CPH (tAUC = 1306 mm × min, 95% CI: 0–3257) and control (tAUC = 1243 mm × min, 95% CI: 0–3418, *P* = 0.966). Data are presented in Fig. 2b.

The questionnaire addressing hunger did not reveal any differences in the feeling of satiety and hunger between CPH (3.4 ± 1.8) and control (3.4 ± 2.3) 180 min after the breakfast (*P* = 0.822). Baseline scores did not differ before each intervention (CPH: 3.7 ± 2.7 and control: 3.0 ± 2.5, *P* = 0.165).

### Gastrointestinal symptoms

There were no reports of adverse gastrointestinal symptoms (e.g. nausea, bloating, stomach pain, constipation, or diarrhea) during the exposure for either CPH or control.

## Discussion

This study revealed no differences in postprandial concentrations of acylated ghrelin after a meal supplemented with CPH compared to control. Thus, we were unable to confirm our hypothesis that a single dose of CPH supplementation before a meal would suppress ghrelin concentrations postprandially, and thereby cause reduced feeling of hunger. Moreover, we found no differences between control and CPH drink in the feeling of satiety or feeling of fullness, as measured by the implemented questionnaires.

In a previous publication, we reported that supplementation with 20 mg CPH per kg body weight before a breakfast meal reduced the postprandial concentrations of insulin compared to control in healthy individuals (14). Although not affecting glucose levels or concentrations of GLP-1, we found that pre-prandial supplementation with one low dose of CPH may beneficially alter the glucose metabolism. An inverse correlation between postprandial insulin concentrations and plasma ghrelin has previously been reported (24), and changes in ghrelin concentration after CPH might affect postprandial insulin secretion. The major effects of ghrelin are linked to mechanisms involved in avoiding starvation and promoting food intake and include stimulation of GH secretion to restrict peripheral glucose uptake, promote lipolysis, and suppress insulin secretion to prevent hypoglycemia (25). Due to this close link between glucose metabolism and appetite control, it can be hypothesized that supplementation with CPH might influence postprandial ghrelin concentrations and the feeling of hunger and satiety.

Our results are partly in line with previous similar single-dose studies; however, few studies are comparable in test material. Most studies investigating the relationship between ghrelin levels after meals with different macronutrient composition have found that a high-protein meal causes the ghrelin levels to be suppressed for a longer time than after the intake of a meal high in carbohydrates (26–31). Furthermore, it causes higher satiety scores postprandially than a meal containing regular or low amount of protein (28, 32). The mechanisms suggested to facilitate these findings include slowing of gastric emptying, increase of plasma insulin, glucagon, ghrelin, CCK, gastric inhibitory polypeptide (GIP), and GLP-1 after a high-protein meal (33).

Only a few studies have reported on the specific acute postprandial effect of a meal with proteins from fish, compared to other protein sources (34–36). A comparison of the effects of isocaloric meals with proteins from beef, chicken, or fish revealed a significantly higher satiety score after the fish meal compared to the other protein sources (34). A study evaluating the effect on satiety when comparing a fish protein meal with a beef protein meal revealed that subjects receiving the fish-meal had lower hunger scores and consumed less energy in the subsequent evening meal (36). In contrast, a study investigating the acute effect of meals based on proteins from cod or veal in combination with carbohydrates high- or low-glycemic index did not find any differences in appetite sensation, energy intake, or postprandial response in glucose, insulin, or ghrelin levels when comparing the two different protein sources (35). Although some previous studies have reported fish proteins to suppress appetite, no effect has this far been reported for the levels of ghrelin. One previous study has reported on the specific hunger-regulating effect of a fish protein hydrolysate from blue whiting (2 g/day) (17). The fish protein hydrolysate was reported to suppress appetite when compared to placebo in a 2-week crossover trial in overweight women. According to postprandial measures after a standardized breakfast meal, it was observed that the fish protein hydrolysate significantly reduced sweet-cravings, as well as plasma glucose levels compared to placebo. This study was based on observations made in both *in vitro* and *in vivo* models, showing that the fish protein hydrolysate was capable of enhancing the secretion of CCK and GLP-1, both hormones contributing to the regulation of energy intake (37).

In the present study, we hypothesized that a potential suppressing effect on appetite and postprandial ghrelin levels would occur due to the CPH containing a high fraction of di- and tripeptides with the branched-chain amino acids leucine and isoleucine. We hypothesize that these peptides work as biologically active substances, which are rapidly absorbed from the gastrointestinal tract and possibly capable of influencing pathways involved in the regulation of appetite. Thus, a single low dose of peptides was administered to the participants. The amount of protein provided was so low that it can be regarded negligible *per se,* compared to the amount of protein provided in the breakfast meal. Several factors could explain the lack of observed effect. First, it is possible that one acute exposure of the low concentration is not enough to induce the wanted effect. A different effect could might have been observed if the participants had taken the CPH supplement daily for a period of time, for instance over a period of 6–8 weeks.

A similar dose of CPH as the one administered in our trial, has previously been reported to increase concentrations of CCK and GLP-1 compared to placebo in a study including 120 overweight individuals given either 1.4 or 2.8 g protein hydrolysate from blue whiting or placebo, for 90 days (18). The fish protein hydrolysate was found to be effective compared to placebo, but no difference in effect was observed between the two doses. This demonstrates a potential effect when CPH is administered orally in doses of approximately 15–20 mg per kg body weight. In our study, the subjects consumed CPH in a dose range from 1.2 to 2.3 g (mean 1.5 g), dependent on body weight. Thus, if an effect was to be observed, this could possibly have been attributed to the presence of bioactive peptides.

The results have to be interpreted taking certain strengths and limitations into account. First, the randomized, crossover design as well as the successful blinding with similar test drink and control is a strength of this study. Furthermore, the adjustment of peptide dose according to the body weight of each participant can be regarded as an improvement in accuracy compared to previous investigations of protein meals and the few studies investigating a marine protein hydrolysate, as this may reduce the effect of variation in body weight. It can be regarded a weakness of the design that the control drink contained casein in equal amounts as CPH, and that we did not include a true placebo without protein. However, as discussed above, the hypothesized effect is attributed to the presence of bioactive peptides and not protein *per se*. Whole casein was chosen as control, so the observed effect should not be simply due to differences in energy and nitrogen content. Casein has previously been shown to not affect glucose metabolism when given in low concentrations (38). We could arguably have investigated the effect of CPH on several different hunger-regulating hormones, for instance, CCK, which has been measured in few other studies investigating the effect of a fish protein hydrolysate on appetite (17, 18). The assessment of appetite could have been improved by using a more comprehensive instrument at all timepoints (39). However, the use of VAS ratings in the evaluation of appetite in healthy subjects is a previously validated method (40). An *ad libitum* lunch meal with subsequent calculations of actual energy intake after CPH and control could have been included for a better and more detailed investigation of appetite. Furthermore, it is possible that the lack of sufficient previous data to perform a power analysis could result in too few included participants to be able to observe an effect. We decided to include 40 participants, a number greater than or equal to previously reported studies on cod protein (12, 13, 23).

Further studies should aim to evaluate the impact of fish protein hydrolysates on different metabolic pathways involved in glucose metabolism and appetite control, such as regulation of different hunger-regulating hormones. In addition to ghrelin, insulin-like peptide 5 (INSL5) is quite recently suggested to be an orexigenic hormone influencing appetite and regulation of food intake (41). Thus, future studies should aim to evaluate the appetite-regulating effect of CPH on several hormones, including INSL5. Assessments of such response can arguably contribute to the expansion of knowledge on the effects of CPH, as well as possibly reveal new preventive and treatment options for overweight and obesity. Based on the current literature, the effect could be more apparent if the fish protein hydrolysate had been given daily over a period of time, and if it had been investigated in a target group of overweight and obese individuals.Thus, the design of future studies should take this into account.

In conclusion, we did not find any effect of a single dose of CPH on postprandial concentrations of acylated ghrelin, or sensations-related appetite in healthy individuals, when compared to control after a standardized breakfast meal.

## Founding

This work was funded by the Norwegian Council of Research (grant number 256684), Haukeland University Hospital, the University of Bergen, Ålesund Hospital and Firmenich Bjørge Biomarin AS.

## Authors

HFD, CJ, TH, IB, JGH, DALH and GAL designed the present study. HFD and CJ conducted the research. IB analysed the ghrelin samples. HFD wrote the manuscript. EL obtained funding and provided administrative, technical and material support. All authors reviewed and approved the manuscript.
